# Repurposing a traditional Japanese method of pest control for wintering pine moths, Komo-trap, for use against summer and winter populations of fall webworms

**DOI:** 10.7717/peerj.9244

**Published:** 2020-06-02

**Authors:** Osamu K. Mikami, Misaki Takamatsu, Rika Yarita

**Affiliations:** Department of International and Regional Studies, Hakodate Campus, Hokkaido University of Education, Hakodate, Hokkaido, Japan

**Keywords:** Fall webworm, Hyphantria cunea drury, Integrated pest management, Komo-trap, Chemical-free, Street tree, Urban greening, Sheet of straw, Low cost

## Abstract

**Background:**

The fall webworm, *Hyphantria cunea* Drury (Lepidoptera: Erebidae), is a widespread invasive species. It is native to North America, ranging from southern Canada to northern Mexico. During and after the 1940s, this pest was accidentally introduced in many parts of Europe and Asia. It has now spread to more than 30 countries. The larvae feed on leaves of a wide range of tree species, including ones used as street trees in cities, causing an increase in urban management cost. Although several pest management methods have been employed, pest damage continues especially in newly invaded areas. In this study, we examined the effect and cost-effectiveness of the komo-trap, traditionally used in Japan to reduce the population of larvae of the pine moth *Dendrolimus spectabilis* Butler (Lepidoptera: Lasiocampidae). This trap, which is safe for people and ecosystems, has not yet been applied to trap the fall webworm.

**Methods:**

In two seasons of 2017, we set komo-traps on street trees in Hakodate City, Japan. We counted the numbers of captured fall webworms compared with controls. We also monitored other species to evaluate any nontarget effects of the trap.

**Results:**

One komo, the material cost of which is about 1.10 USD, captured 43.8 fall webworms on average in summer and 27.2 in the fall. The values were significantly larger than those of the controls, which were 0.07 in summer and 0.14 in winter. Bycatch of other species was minimal in summer, whereas in the fall one komo, on average, caught 10.7 woodlice *Porcellio* sp. or spp. (Isopoda: Porcellionidae).

**Discussion:**

The komo-trap is effective in capturing fall webworm. The cost performance of the trap is very favorable, and the nontarget effects can be reduced by using the trap in summer only. The komo-trap would complement other control methods such as tree pruning. Because its cost is low, we recommend that the komo-trap be introduced as a larger-scale trial.

## Introduction

The fall webworm, *Hyphantria cunea* Drury (Lepidoptera: Erebidae), is native to North America, ranging from southern Canada to northern Mexico. During and after the 1940s, this pest was accidentally introduced in many parts of Europe and Asia (Ge et al., 2019; Schowalter & Ring, 2017). It is now found in more than 30 countries worldwide. The number of generations per year of the pest depends on temperature and race. When it comes to two generations per year, overwintering pupae emerge as adult moths in early summer. Adult females lay egg masses on the underside of leaves. In summer, larvae hatch from the eggs and create silken web nests. They expand the web nests to enclose and feed on leaves. In mid-summer, mature larvae leave the web and wander alone looking for a pupation site. The larvae choose dark, moist sites, such as in bark crevices, under stones, in the duff, or just beneath the soil surface (Drooz, 1985). They emerge as first-generation moths. Female moths then lay eggs in late summer. The second-generation larvae hatched from the eggs, damage trees in fall and pupate before winter.

The larvae feed on the leaves of over 400 tree species, including street trees in urban areas (Schowalter & Ring, 2017). In native areas, their activity does not cause considerable damage because there are natural enemies (Edosa et al., 2019). However, in newly invaded areas, their feeding activity affects the greening and beautification of urban environments and, consequently, causes an increase in management costs for cities. Future climate change will affect the distribution of this pest, resulting in tree damage in new areas (Ge et al., 2019). Several pest management methods exist, for example, physical removal, biocontrol, insecticides, and traps (Cui & Zhu, 2016; Edosa et al., 2019). Novel options for controlling the pest population are needed to develop integrated pest management strategies.

In this study, we examine the effect of the komo-trap on the fall webworm. The komo trap is a traditional trap used in Japan to reduce the population of larvae of the pine moth, *Dendrolimus spectabilis* Butler (Lepidoptera: Lasiocampidae). In Japan, pine trees, *Pinus thunbergii* Parlatore (Pinales: Pinaceae) and *P. densiflora* Siebold et Zucc, are often planted as ornamental garden trees. To protect pine trees from pine moths, the komo-trap was developed in the Edo period (1,603–1,868). Gardeners wrap a komo, a straw mat, around the tree trunk before winter, which provides pine moth larvae an apparently safe place to overwinter. Early in the following spring, before the larvae emerge, the gardeners can easily eliminate the larvae by removing and burning the komos along with the larvae contained in it. Such an inexpensive trap with simple structure can be used for reducing and/or monitoring pest insects (e.g., Hegazi et al., 2009; Daniel, Mathis & Feichtinger, 2014). The komo-trap can be used for mass trapping. In general, mass trapping attracts target species by chemical lures, such as sex and aggregation pheromones and/or food (El-Sayed et al., 2006). However, the komo-trap attracts pests by providing an overwintering habitat.

The komo-trap has two advantages. First, its simplicity—anyone can install one, and no specific tools are required. Second, because no chemicals are released in the environment, there are no concerns about adverse impacts on the ecosystem (Kohler & Triebskorn, 2013) or on humans, especially infants and children, who are more sensitive to chemical toxicity than are adults (Bruckner, 2000).

Although we could find no reports of attempts to apply the komo-trap to fall webworm, we assumed that it had the potential to control this species because the larvae also choose dark, moist sites to pupate in their thin cocoons. Here, we elucidate the effects of the komo-trap when used against fall webworm, and its potential nontarget effects. We also discuss its cost-effectiveness.

## Materials & Methods

### Study area

The survey was conducted in Hakodate City, Hokkai-do, Japan. By assuming where the trap will be installed, we conducted the study on street trees. We selected seven roads bordered with numerous street trees from within a range of approximately 1 × 1 km (the center of the study area was 41°47′40.7″N 140° 44′29.7″E) and focused on three tree species, Japanese rowan *Sorbus commixta* Hedl. (Rosales: Rosaceae), American sycamore *Platanus occidentalis* L. (Proteales: Platanaceae), and Manchurian ash *Fraxinus mandshurica* Rupr. (Scrophulariales: Oleaceae), all of which are vulnerable to the fall webworm. The numbers of trees of the three species planted in the study area were 130, 355, and 93, respectively (Table 1). We obtained permission from the Hakodate City Office to perform all experiments.

**Table 1:** Schedules, number of trees with komos wrapped, and the number of fall webworms counted for the summer and fall experiments.

		Experiment					
		Summer			Fall		
Schedule	checking nest webs	Jul 10,11,12			Sep 4,5		
	komo installation	Jul 24,25,26,27			Sep 24, 26, 27		
	komo removal	Aug 3, 4, 7, 8			Oct 31; Nov 2, 6, 7		
Tree species		Rowan	Sycamore	Ash	Rowan	Sycamore	Ash
Number of trees	planted	130	355	93	130	355	93
	with nest webs	22	14	11	26	183	72
	komo-wrapped	21	12	10	12	20	18
Number of fall webworms trapped	pre-pupae larvae	57	71	8	87	138	249
	pupae	907	610	229	77	62	679

### Komo installation

**Figure 1:**
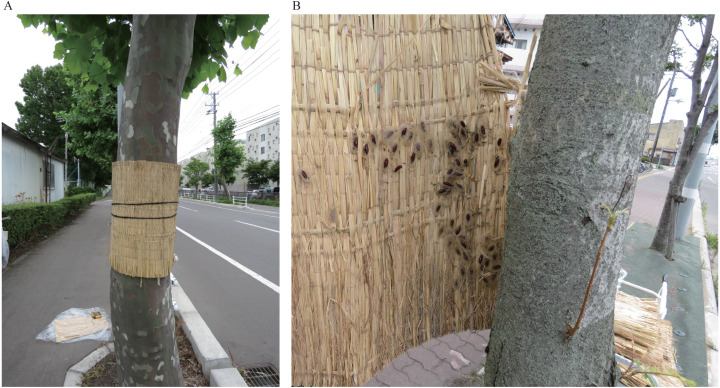
Photographs of (A) a komo-trap on the trunk of an American sycamore and (B) pupae captured in a komo-trap set on the trunk of a Manchurian ash.

The fall webworm usually has two generations per year in the study area and damages street trees in summer and fall. We set komo-traps in both seasons in 2017. Hereafter they are called the summer experiment and the fall experiment (Table 1). We checked all the trees for the presence or absence of webs and wrapped a komo around the trunk of some of the trees in which one or more nest webs were found. We did not wrap a komo on the others trees because we used them as controls. Each komo was 0.5 m high and 1.7 m wide. When the komo width was greater than the circumference of the trunk, the redundant section was cut off. Each komo was secured by two hemp ropes and positioned so that the top edge of the komo was 1.5 m above the ground (Fig. 1). In three cases, one or more branches were present on the trunk coinciding with the intended wrapping position; therefore, the komo was positioned lower down. We measured the diameters at breast height (1.3 m above the ground) of the trees wrapped with the komos to examine the relationship between tree thickness and the number of captured fall webworms. If there is a relationship between them, it may be possible to select trees suitable for installing the trap.

In the summer experiment, the komos were removed 10 or 12 days after installation, whereas in the fall season, they were removed over 1 month after installation (Table 1). In the summer experiment, if they were installed for a longer time, there would be a risk of the pests emerging before the removal of the komos. In the fall experiment, there was no such risk. In addition, the timing of pupation of the pests varied in the fall experiment.

### Counting fall webworms and other species

When komos were removed, we counted the numbers of fall webworm pupae and pre-pupal larvae attached either to the komos or to the trunk where the komos were removed. We also counted the number of other species larger than about five mm in size found on the komos and on the trunk beneath to evaluate any nontarget effects of the komo-trap. In the summer experiment, when we removed the komos, many such species escaped because they were hiding between the komo and the trunk; these were omitted from the counting because they had not been removed from the environment by the komo-trap. The pupae of the fall webworms were collected, transported to our laboratory, and sexed on the basis of their abdominal patterns according to the method of Loewy et al. (2013) using a stereo microscope.

### Control

We conducted control experiments, “control” meant “under a situation where there are no komos.” In other words, the control experiments could reveal how many fall webworms were captured by the komo-trap. As the control for the fall experiment, we counted the number of larvae on the trunks of trees with nest webs but without a komo (the fall control). The range of trunks, the number and species composition of the trees, and the period we conducted the counting were also the same as for the fall experiment. The control for the summer experiment (the summer control) was conducted in the same way; the range of trunks and the number and species composition of the trees were also the same as those for the summer experiment. But it took place in the summer of 2018, i.e., the year following the summer experiment. Checking web nests was conducted from July 23, 2018 to July 25, 2018, and counting fall webworms was performed from August 5 to August 8, 2018. This is because the number of trees damaged by the fall webworm in the summer of 2017 was insufficient for a control to be conducted then; almost all the damaged trees were counted in the summer experiment.

### Statistical analysis

We used Brunner–Munzel’s test (Brunner & Munzel, 2000), which does not assume normality and homoscedasticity, to analyze the difference in the number of fall webworms counted between the experiment and the control in each season. We used a chi-square test to analyze whether sex ratio of pupae differed from 1:1. We used Pearson’s product–moment correlation test to test the relationship between host tree diameter at breast height and the number of fall webworms counted. All statistical analyses and graph generations were performed by using R (version 3.4.2) (R Core Team, 2017). R packages “brunnermunzel” (Ara, 2019) and “beeswarm” (Eklund, 2016) were also used.

## Results

**Figure 2:**
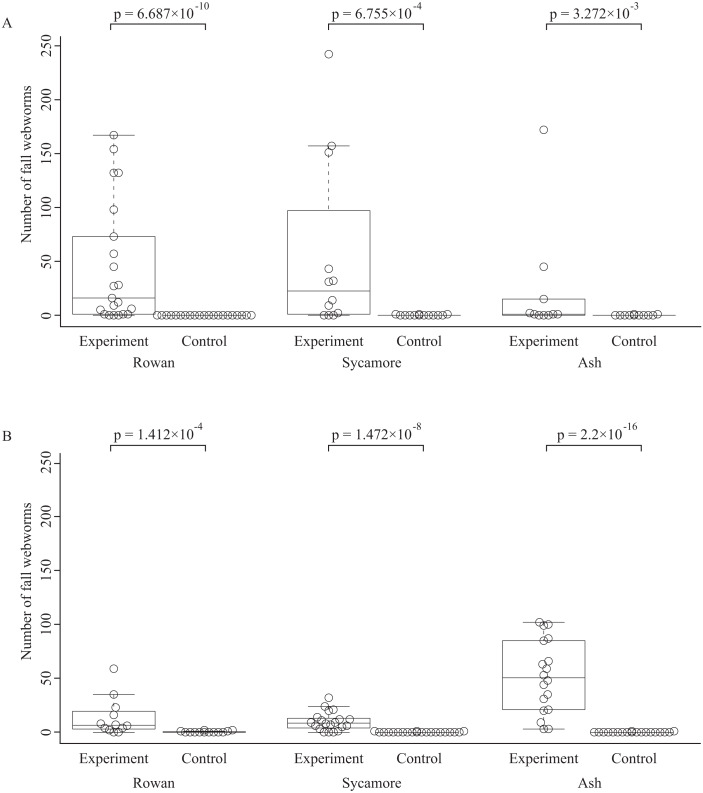
Box plots and beeswarm plots of the number of fall webworms counted in (A) summer and (B) fall. The *p*-values are obtained from Brunner–Munzel’s test. They are not controlled for multiplicity.

**Figure 3:**
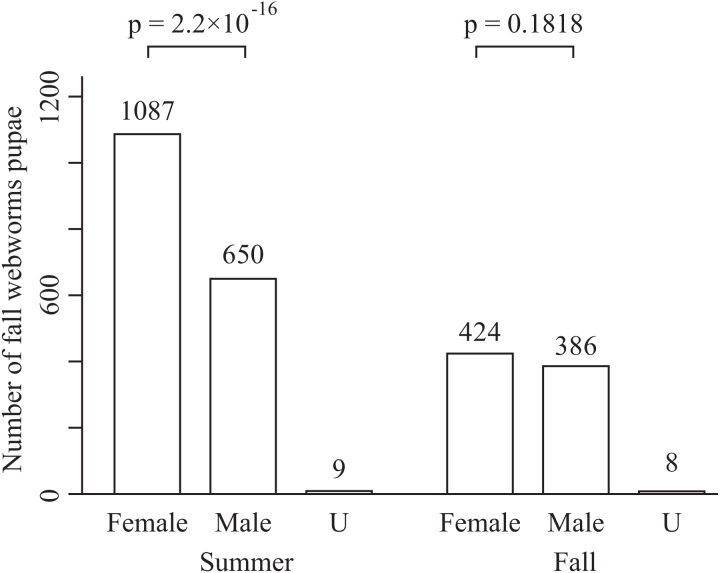
Number of male and female fall webworm pupae captured in the summer experiment and the fall experiment. “U” means unidentifiable. The *p*-values are obtained from chi-square test. They are not controlled for multiplicity.

The total numbers of fall webworms captured were 1,882 in the summer experiment, 3 in the summer control, 1,292 in the fall experiment, and 7 in the fall control. Some komos captured zero or only a few fall webworms. In both seasons, and for each of the tree species, the number of fall webworms counted was significantly greater in the experiment than in the control (Fig. 2). We collected 1,746 fall webworm pupae in the summer experiment and 818 in the fall experiment. The rate of females was 0.62 (1,087 females/1,746 pupae) in the summer experiment and 0.52 (424 females /818 pupae) in the fall experiment. The sex ratio was significantly biased towards females in the summer experiment, but there was no significant difference in the fall experiment (Fig. 3).

Regarding nontarget species, fewer were captured in the summer experiment than in the fall experiment (Table 2). All the Malacostracans captured were woodlice (*Porcellio* sp. or spp.).

**Table 2:** Counts and Family-level identification of nontarget organisms captured in komo-traps during the summer and fall experiments.

Class	Order	No. of individuals	
		Summer	Fall
Arachnida	Araneae	5	14
Chilopoda	Scutigeromorpha	3	1
Diplopoda	Unidentifiable	1	0
Malacostraca	Isopoda	1	534
Insecta	Dermaptera	14	1
	Blattodea	1	0
	Hemiptera	3	1
	Coleoptera	3	0
	Diptera	12	3
	Lepidoptera	1	0

**Figure 4:**
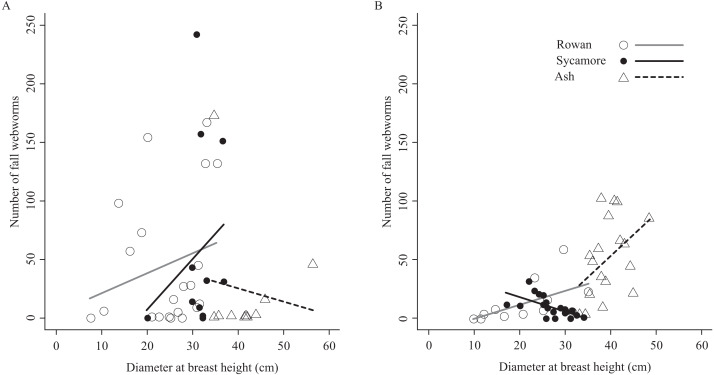
Relationships between host tree diameter at breast height and the number of fall webworms counted in (A) the summer experiment and (B) the fall experiment.

The relationship between the number of fall webworms captured and the tree diameter at breast height was inconsistent (Fig. 4). There were differences according to the tree species and the season. However, excluding these, a weak trend was apparent (Pearson’s product–moment correlation, *r* = 0.198, *t* = 1.9249, *df* = 91, *p*-value = 0.05737); the thicker the tree, the more fall webworms were captured.

The cost-effectiveness of komo-traps in the summer experiment was estimated as follows. The material cost of one komo-trap is 121 JPY, which includes a straw mat and two hemp ropes but excludes the labor cost (121 JPY is approximately 1.10 USD or 0.98 EUR at 2019 yearly average exchange rates). One komo-trap, on average, captured 43.8 fall webworms (1,882 individuals/43 komos). Of these, on average, 27.2 were female (43.8 fall webworms × 0.62 female biased sex ratio). Therefore, one komo-trap exterminated 27.2 × *r*_*s*_ individual females that would have laid eggs in the future, where *r*_*s*_ is the survival rate of the first-generation females from pupae to after egg laying. There are no accurate data on *r*_*s*_ in the field. When *r*_*s*_ = 0.5, 13.6 females that would contribute to population growth were captured for 1.10 USD. Similarly, in the fall experiment, one komo-trap captured 25.8 individuals (1,292 individuals/50 komos) including 13.4 females (25.8 fall webworms × 0.52 female biased sex ratio). One komo-trap exterminated 13.4 × *r*_*w*_ females that would have laid eggs in the future, where *r*_*w*_ is the survival rate of the second-generation females from pupae to after egg laying the following spring.

## Discussion

Some komos did not capture any fall webworms, whereas some captured only a few fall webworms (Fig. 2). This may have been because the fall webworms were killed by predators or parasites. In addition, before we removed komos in the fall experiment, Hakodate City Office carried out normal, regular pruning, and lopped some branches off all the American sycamores, which may have depressed counts in the fall experiment.

One komo-trap, on average, captured 27.2 females in the summer and 13.4 females in the fall. The captured fall webworms were biased to females in the summer but not in the fall (Fig. 3), a difference for which the cause is unclear. The sex ratio of the studied population in summer may be skewed to females. Alternatively, there may be physiological or behavioral differences between females and males, which may result in more females entering the komo-trap than males in summer. As another possibility, we may have obtained the current results because of lower temperatures. The rate of generation of pre-pupae larvae was higher in the fall experiment (Table 1). This was because some mature larvae were not able to pupate possibly because of the lack of suitable temperatures. Such phenomenon often occurs in colder years in the study city. These larvae may be biased towards females because the larval period of female is longer than that of male (Gomi et al., 2005).

The high proportion of females is beneficial from a trapping viewpoint because females directly contribute to population growth. Capturing many females or at least capturing equal proportions of males and females is an advantage of the komo-trap. This contrasts with pheromone traps that, in general, capture males rather than females because the attractant is usually based on the female-produced pheromone. Pheromone traps disrupt mating of a target species (Trematerra, Ferrario & Binda, 1993; Yamanaka, 2006) and indirectly suppress its population growth. Therefore, a large number of traps must be installed to reduce populations. Conversely, the komo-trap can suppress population growth by capturing females directly.

Without considering seasons and tree species, weak relationships were obtained between the thickness of trees and the number of the fall webworms trapped (Fig. 4). One possible reason may be that larger trees have more larvae of the fall webworm because of the availability of sufficient food. It may also be because a thick trunk is more likely to be encountered, by chance alone by mature larvae wandering looking for a pupation site. If the cost of selecting trees is small, komo-traps may be installed preferentially on larger trees.

The komo-trap has some nontarget effects (Table 2), especially in the fall, when the 50 komo-traps caught 534 woodlice, a number less than half that of captured fall webworms. As a countermeasure, installing the komo-trap only in summer would significantly reduce this nontarget effect (Table 2). One advantage of the komo-trap is that nontarget effects can be accurately evaluated.

The cost performance of the komo-trap is very favorable. One komo (material cost is 1.10 USD) captured 27.2 × *r*_*s*_ individual females in the summer and 13.4 × *r*_*w*_ individual females in the fall, most of which would have laid eggs in the future. The second of the values, 13.4, may have been an underestimate because of the effects of tree pruning during the experiment, but even so, the cost performance in the summer (i.e., the cost performance of managing first-generation larvae) may be higher than that in the fall. This is because *r*_*s*_ is generally larger than *r*_*w*_, as the summer season is shorter than the winter season (Itô & Miyashita, 1968). In other words, most pupae in the fall die naturally and contribute little to population growth. If *r*_*s*_ = 0.5 and one female lays 500 eggs, one komo-trap set in summer will eliminate 27.2 × 0.5 × 500 = 6,800 future larvae in the fall. It is possible that other methods cannot eliminate 6,800 larvae for 1.10 USD. The number of eggs laid by a female varies according to different studies. For example, Loewy et al. (2013) reported 484.2 (range: 34–830), Yearian, Gilbert & Warren (1966) reported 650 (range: 300–1,350), and Itô, Shibazaki & Iwahashi (1969) reported of 425–1,050. Here, it is set at 500 so as not to be overestimated. It is also challenging to achieve the same effect as the komo-trap by capturing larvae before they become pupae. Assuming that the survival rate from early larva to pupa is 0.02 (Itô & Miyashita, 1968), to achieve the same effect as the komo-trap (i.e., one komo-trap eliminates 43.8 pupae) by eliminating larvae, it would be necessary to eliminate 43.8∕0.02 = 2, 190 larvae, a task that would also be difficult to accomplish for 1.10 USD.

The komo-trap could be even more effective if combined with existing control methods. Consider, for example, its combination with tree pruning. Tree pruning physically removes fall webworm larvae and reduces tree damage. Then, the komo-trap could play a supplementary role by capturing pupae that survive pruning. In addition, in the case that high pruning affects the productivity of the trees, one could reduce pruning and instead set many komo-traps. The komo-trap may also be combined with biological control using natural predators that target pupae (e.g., Yang et al., 2015; Yang, Wei & Wang, 2006). If the natural enemies preferentially attack the fall webworm pupae that are not hidden in komo-traps, the komo-trap and the biological control methods would be complementary. On the other hand, the komo-trap may not be compatible with traps designed to capture adult moths (Trematerra, Ferrario & Binda, 1993) because komo-traps decrease the number of moths and may reduce the cost performance of the traps for them.

This study has a problematic issue and few limitations. The problematic issue is the schedule of the summer control; the summer control was conducted in the year following the summer experiment because the number of trees damaged by the pest was insufficient. It should be noted that the damage to trees by fall webworms varies annually. However, usually, fall webworm larvae do not pupate on the surface of a tree trunk without grooves; even in the case of ordinary bark, they pupate in dark and moist places. This was demonstrated by the fall control; only 7 fall webworms were found on trunks of 50 trees. All fall webworms counted in the controls were found in deep crevices and cavities in the trunks. Therefore, without komos, it is impossible to capture larvae of the fall webworm on the trunks in large numbers. Hence, we believe that komo is effective in capturing fall webworms.

The first limitation is that we did not research any adverse effects the komo-trap may have had on the trees because of wrapping komos on the trunks. However, as no reports exist, it is assumed that there is no significant effect because pine trees, the original tree species for which komo-traps are used, are not harmed. The second limitation is that we did not directly research whether the komo-trap protects street trees from the fall webworm. To research this aspect would require experiments on a larger-scale. Adult fall webworms could fly from outside the study area to damage the street trees. Fall webworm adult males may travel hundreds of meters per day (Yamanaka, Tatsuki & Shimada, 2001; Zhang & Schlyter, 1996), whereas the maximum flight for females has been reported as 188 m (Suzuki & Kunimi, 1981).

Despite these limitations, we recommend that the komo-trap be introduced as a larger-scale trial because its initial cost is low. The effects of the trap could vary by region and location, but if the traps are not proving effective, they can simply be removed. If the traps were effective, they could be combined with current methods that may lead to a reduction in the total cost of controlling the fall webworm.

Finally, we make four proposals to increase the effectiveness of the komo-trap: (1) since the overwintering generation has high natural mortality, it is likely to be more effective to focus on using the traps on the remaining generations; (2) komo-traps should be set on trees already damaged by fall webworm or on nearby trees because the fall webworm caterpillars do not migrate long distances; (3) the trap is suitable for all trees, and trees of varying size; and (4) the komos must be removed at the appropriate time (1 or 2 weeks after installation in case of the remaining generations); otherwise, they would act as a safe habitat for the fall webworm, which may result in an increase in their emergence rate.

## Conclusions

In this study, we have shown that the komo-trap is potentially effective in reducing the population of fall webworm. The trap is safe for people, is low cost, and has few adverse effects on other species. The komo-trap could become part of an effective integrated pest management program (Kogan, 1998) for the fall webworm, especially in urban areas where the use of insecticides is undesirable.

Two aspects need to be clarified in future research. First, it is necessary to assess the effect of the komo-trap when combined with other control methods. Second, for areas where the raw materials for making komos (sheets of straw) are not available, it would be beneficial to test alternatives that provide the same effect, something relatively thin and flexible, with a rough surface, and that is light-tight to provide a dark environment.

##  Supplemental Information

10.7717/peerj.9244/supp-1 Supplemental Information 1 Raw data

## Additional Information and Declarations

### Field Study Permissions

The following information was supplied relating to field study approvals (i.e., approving body and any reference numbers): Field experiments were approved by Hakodate City Office.

### Data Availability

The following information was supplied regarding data availability: The raw measurements are available in the Supplementary File.
